# Prevalence, Risk Factors, Awareness, and Treatment and Control of Hypertension in Mafia Island, Tanzania

**DOI:** 10.1155/2016/1281384

**Published:** 2016-07-20

**Authors:** M. S. Muhamedhussein, Z. I. Nagri, K. P. Manji

**Affiliations:** ^1^Regency Medical Center, P.O. Box 2029, Dar es Salaam, Tanzania; ^2^Shree Hindu Mandal Hospital, P.O. Box 581, Dar es Salaam, Tanzania; ^3^Department of Pediatrics and Child health, Muhimbili University of Health and Allied Sciences, P.O. Box 65001, Dar es Salaam, Tanzania

## Abstract

*Introduction*. The prevalence of hypertension in Africa ranges from 29.7% in Cameroon to 47% in South Africa. Only 10% receive treatment in Cameroon while 32% are on medications in Ghana. Control rates vary from 0.4% to 16.8%. This study was done to assess prevalence, risk factors, awareness, treatment, and control of hypertension in Mafia Island, Tanzania, which has never been documented before, so that necessary interventions can be undertaken accordingly.* Methodology*. Data was collected through questionnaires and anthropometric measurements were taken. Descriptive statistics were done and potential correlations were analyzed.* Results*. Out of 570 adults who were included in the study, 154 (27%) were aged 41–50 and the male-to-female ratio was 1 : 1.05. Almost half (49.5%) of the participants fit into the criteria of hypertension. Out of the 118 participants who were aware of having hypertension, 68 (57.6%) were currently taking medication. From those taking medication, only 14 (20.6%) had controlled hypertension.* Conclusion*. This study tried to show the extent of hypertension and find out risk factors which could explain the high prevalence of hypertension. This is very alarming and a dire need to raise awareness through health education, availability of screening, and treating and follow-up should be given priority.

## 1. Introduction

Hypertension is a rapidly emerging disease worldwide and contributes highly to morbidity and mortality. Risk factors not only include obesity, sedentary lifestyle, and age but also genetics and environmental factors contribute to it. Hypertension is usually asymptomatic and the patient presents to the hospital with one of the complications especially in developing countries where very minimal importance and minimal resources are allocated to noncommunicable diseases.

A study showed that 80% of deaths that result from noncommunicable diseases occur in the developing world [1]. Another study has predicted an increase of 80% in the number of hypertensives by 2025 [2]. Studies from South Africa have shown that obesity and hypertension are the most prevalent cardiovascular risk factor [3]. As a result, the most common presentation with chronic heart failure is due to hypertensive heart disease [4]. By 2030, 85% of the 23 million cardiovascular deaths projected will be in low and middle income countries [5].

In Cameroon, 29.7% of all participants in a study had hypertension [6] while it was a staggering 47% among females in South Africa [7]. Treatment of those who were known to have hypertension was a mere 10% in Cameroon [8] and 32% in Ghana [9]. Out of those using medicines, only between 0.4% and16.8% had optimal control [7, 8]. In another study conducted in Cameroon, 47.5% of people had hypertension, from which only 31.7% were aware of their status [10]. A study done in Kilimanjaro showed prevalence of hypertension to be 30% in males and 28.6% in females of which under 20% of the patient with hypertension were aware of their status. Only 10% were on medication and less than 1% had optimal controlled blood pressure [8].

Health education is extremely vital in health care system, particularly in chronic conditions such as hypertension, which can be prevented, delayed or controlled with the help of medications and lifestyle changes. Developing countries, due to the high burden of infectious diseases, are far behind the ideal health awareness and education in their population. For the government and health care workers to put emphasis on a particular disease or diseases, the burden of that particular disease should be known. This study aimed at providing a baseline so that the governement can allocate its resources according to the need to be able to reduce morbidity and mortality and to improve health of the people.

## 2. Methodology

### 2.1. Study Design and Setting

This cross-sectional study was done in Mafia Island, Tanzania. Mafia Island is part of the Tanzanian Spice Islands, together with Unguja and Pemba. According to the 2002 Tanzania census, the population of the Mafia District was 40,801 [11]. The economy is based on fishing and subsistence agriculture. Mafia's infrastructure is poor: it has electricity only in the district capital and in Utende, the main tourist area. There are few houses which have running water. The vast majority of Mafia's population is extremely poor. The major cash crop, coconuts, has a declining price on both world and local markets. Mafia has no newspapers, bookshops, or libraries, and people are primarily dependent upon radio for information about the world beyond the East Coast of Africa. However, modern telecommunications are beginning to be available [12].

### 2.2. Sampling Method and Data Collection

The study used convenience sampling method and achieved a sample size of 570 nonpregnant adults. An eye and medical camp was organized in Mafia Island in October 2011. This was held at a primary school compound in which there was prior notice to the local people to raise awareness of the medical camp. The questionnaire was filled by medical students, medical personnel, and research assistants whereas measurements were done by medical students and medical personnel only. The information on demographics, smoking habits, alcohol consumption, family history of hypertension, and personal medical history (only diagnosis made by a doctor was considered) including drug treatment was collected by using a questionnaire in Kiswahili. The questionnaires were tested within Dar es Salaam before the research. Participants with high blood pressure were considered to be aware of their status if they answered “yes” to the question “Do you suffer from hypertension?” Treatment of hypertension was defined by the use of blood pressure lowering medications.

### 2.3. Measurement of Blood Pressure and Anthropometry

Blood pressure was measured using aneroid sphygmomanometer (RIESTER brand) while the subject was seated and relaxed with their hand at the level of the heart. People who had eaten food, smoked cigarette, or used alcohol within 30 minutes were asked to rest for 30 minutes before measurements were taken. A standard size of cuff was used.

Anthropometric measurements (weight and height) of the sample were taken, body mass index thereafter calculated by using weight (Kg)/height (M)^2^. Weight was taken using a regular weighing machine after removal of shoes and excess weight in pockets while height was taken using a measuring board resting on the wall. Weight was calculated to nearest 0.5 kg and height to nearest 1 cm.

### 2.4. Definition of Terms

Blood pressure was classified according to the Joint National Committee for hypertension (JNC VII). It was classified as normal blood pressure if systolic blood pressure was between 90 and 119 mmHg and diastolic blood pressure was between 60 and 79 mmHg, prehypertension (mildly elevated) when systolic blood pressure was 120–139 mmHg and diastolic blood pressure was 80–89 mmHg, Stage 1 hypertension (moderately elevated) when systolic blood pressure was between 140 and 159 mmHg and diastolic blood pressure was between 90 and 99 mmHg, and Stage 2 hypertension (severely elevated) when systolic blood pressure was more than 160 mmHg and diastolic blood pressure was more than 100 mmHg [13]. Hypertension was considered to be controlled among treated individuals when systolic blood pressure was less than 140 mmHg and diastolic blood pressure was less than 90 mmHg.

Body mass index was categorized according to World Health organization and classified as underweight if BMI < 18.5 kg/m^2^, normal if it was between 18.5 kg/m^2^ and 25.0 kg/m^2^, and overweight if it was from 25.1 kg/m^2^ to 30 kg/m^2^. The participants were categorized as obese when body mass index was between 30.1 kg/m^2^ and 40 kg/m^2^ and morbidly obese if BMI was more than 40 kg/m^2^ [14].

### 2.5. Statistical Analysis

Descriptive statistics were used to present tables. Data analysis was done from the processed data first by running frequencies, making comparison tables with regard to blood pressure and *p* value was calculated using two-variable Chi-square test. Univariate analysis of variance was also done. Data was entered and processed in SPSS version 17. In this study, *p* value of ≤ 0.05 was considered to be significant.

### 2.6. Ethical Considerations

Permission to conduct the study was obtained from the school of medicine at Muhimbili University of Health and Allied Sciences (MUHAS). Also, permission was sought from responsible authorities in Mafia Island.

## 3. Results

Twenty-seven percent of the participants were between 41 and 50 years of age. The mean age was 44.53 with standard deviation of 13.92. There were almost equal males and females with 50.9% of them being females and 49.1% being males. The majority (53.3%) of the participants had primary level education while 28.1% had no formal education.

Almost a third (32.6%) of the participants had mildly elevated blood pressure and 24.6% had moderately elevated BP while 24.9% of the participants had severely high blood pressure (Table 1) and hence that makes a total of 49.5% who qualify to be called hypertensives according to American Heart Association.

Out of the 570 participants, 46 (8.1%) were current smokers from which 24 smoke <5 cigarettes a day, 18 smoke between 5 and 15 cigarettes a day, and 4 smoke more than 15 cigarettes a day. A few (6.3%) of the participants consume alcohol. Out of these, 77.8% (28) consume alcohol less than 2 times a week, 16.7% (6) consume alcohol between 2 and 4 times a week, and 5.6% (2) consume alcohol more than 4 times a week.

Out of the 118 patients who were aware of having hypertension, only 68 (57.6%) were currently taking medication. The rest were not taking medications mainly due to unavailability of antihypertensives or unaffordability of these drugs (Table 1). Out of those taking medicines, only 14 (20.6%) had controlled blood pressure while 54 (79.4%) had uncontrolled hypertension.

## 4. Discussion

### 4.1. Prevalence and Awareness

In this initial screening, moderate to severe high blood pressure was seen among 49.5% of the participants. In males, high blood pressure was seen among 52.1% (146) participants while in females it was at 46.9% (136) though this was not statistically significant (*p* value 0.21). Compared to the prevalence of hypertension in Africa, that ranges from 29.7% in Cameroon to 47% in South Africa and the studies done in Dar es Salaam and Kilimanjaro in which prevalence of hypertension was shown to be 30% males and 28.6% females, this is alarmingly high. Despite the limitation of few observations, the cut-off of 140 mmHg systolic pressure and 90 mmHg diastolic pressure is still very crucial.

Of those who had hypertension, only 26.6% were aware of their diagnosis which is a bit higher compared to the study in Dar es Salaam that showed just under 20% of hypertensive subjects were aware of their diagnosis. This may be higher but it is still very low compared to the ideal situation.

### 4.2. Risk Factors

The reason behind this alarming prevalence could be not only the sedentary lifestyle in Mafia Island, diet that includes large amount of salt and fish, but also the low level of education and poor and substandard health facilities. In analyzing the relation between high blood pressure and level of education, it was very significant (*p* value < 0.05). This fact can be reflected by a very high percentage (73.3%) of the participants who did not know if they had hypertension or not (they had never measured). This was particularly disturbing since male and female participants were almost equal and monitoring blood pressure in pregnant women is mandatory. Two-thirds (194 of the 290) of the women had never got their blood pressure measured.

Participants who were underweight had prevalence of high blood pressure at 15.4% while those who had normal BMI had high blood pressure prevalence of 40.2%. Hypertension was seen in 56.2% of overweight people and 65.3% of obese participants. All morbidly obese individuals had high blood pressure. The relation between BMI and high blood pressure was also very significant (*p* value < 0.05).

High blood pressure was almost only (95%) seen in participants above 31 years. Analysis showed there was very significant relation between age and high blood pressure (*p* value < 0.05). High blood pressure was seen among 26.1% of smokers and 51.5% of nonsmokers. The relation between smoking and high blood pressure was very significant (*p* value < 0.05). The blood pressure was elevated in 49.9% of people who used alcohol and 49.3% of those who did not use alcohol. This was not significant (*p* value 0.948). There was also positive corelation between family history and high blood pressure (*p* value < 0.05). The reason of participants being unaware would be a wide variety: from ignorance, not having right information, poor health care seeking behaviors, and unavailability of resources.

### 4.3. Treatment and Control

Out of these 118 patients who were aware of having hypertension, 68 (57.6%) were currently taking medication. This was very high compared to the study that showed 10% receive treatment in Cameroon while 32% are on medications in Ghana and also the Dar es Salaam study that showed approximately 10% reported receiving treatment. Out of the 118 participants who were aware of having hypertension, only 34 (28.8%) had controlled blood pressure (systolic blood pressure < 140 mmHg and diastolic blood pressure less than 90 mmHg). Twenty-four (20.3%) had systolic blood pressure between 140 and 160 mmHg and diastolic blood pressure between 90 and 99 mmHg while 50.8% had severely high blood pressure (systolic pressure > 160 mmHg and diastolic pressure more than 100 mmHg). This may be due to various reasons, among which the most likely would be unavailability of medications which was a complaint that was very often received during the research data collection. Noncompliance of the medicines could also contribute to the poor control and this can be associated with level of education. Out of those hypertensives using medications, only 20.7% had optimal and controlled blood pressure whilst the majority (79.3%) had uncontrolled blood pressure. In comparison, 60% of hypertensives who were not taking medications regularly had controlled blood pressure. However, this was statistically insignificant (*p* value 0.21). This can also be explained by poor adherence to medications or irregular intake. The potential of myocardial infarction, stroke, and poor quality of life reflected from this statistics is very high. Other contributing factors may be lack of physical activity, unhealthy diet, poor awareness, and suboptimal facilities. In verbal discussions with the participants, it was noted that in Mafia there were significant number of adults with stroke but they were treated at home with alternative medications.

### 4.4. Potential Limitations and Strengths of Our Study

Participants were those who attended the eye and medical camp that was well publicized. Only patients who could come by themselves were included in the study, while those sick and debilitated people could not make it. Therefore, those with complications of hypertension and diabetes mellitus were not included; thus, there was selection bias. It remains, however, that participants in the study could still differ from the general population by being more or less health conscious and having less severe disease, for example, which could affect some parameters in the study.

The study used a cross-sectional design, which precludes any causality inference.

Diagnosis of hypertension was based on blood pressure measurements during a single encounter, which is variant with guidelines recommendations for hypertension diagnosis using two measurements recorded on three separate occasions [15]. This may have overestimated the prevalence of hypertension and underestimated the level of blood pressure control. This, however, would not affect comparisons with previous epidemiological studies that have used similar measurement methodology. Diagnosis of hypertension among those with no history of hypertension in our study was based on a single BP measurement. A sensitivity analysis suggests that this approach could inflate the crude prevalence by about 5.4% and 4.6% among those not known to have hypertension prior to the survey and at the total population level, respectively [10]. Nevertheless, even after accounting for this possible effect, our estimates would still be within sampling variation of the figures previously reported in this setting.

The strengths of this study include the large sample of the general population and the rigorous collection of various lifestyle factors, medical data, and blood pressure measurements by trained medical personnel according to standardized protocols. In addition, our sampling approach provides evidence that simple announcements through mass media can attract from the community a large number of individuals at risk of undiagnosed hypertension.

## 5. Conclusion

In this survey in Mafia Island, almost half of the participants had moderate to severe hypertension, most of whom were unrecognized. Together with that, those who were aware of their disease were also assessed for control of the situation and this study showed their control was not adequate. This is very alarming and therefore shows a need to raise awareness and availability of screening, treating, and follow-up. Furthermore, this study can act as a baseline for more studies to be undertaken and more evidence to be gathered.

## Figures and Tables

**Table 1:** Prevalence, risk factors, awareness, and control of blood pressure.

Blood pressure	Normotensive	Hypertensive	*p* value
*Gender*			
Male	134 (47.8%)	146 (52.2%)	0.210
Female	154 (53.1%)	134 (46.9%)	

*Age*			
<40	150 (73.5%)	54 (26.5%)	<0.05
40–50	78 (50.3%)	76 (49.3%)	
>50	60 (28.3%)	152 (71.7%)	

*Educational status*			
No formal education	54 (33.8%)	106 (66.2%)	<0.05
Primary	162 (53.3%)	142 (46.7%)	
Secondary	64 (68.8%)	29 (31.2%)	
College	8 (66.7%)	4 (33.3%)	

*Family history*			
Yes	88 (44.9%)	108 (55.1%)	<0.05
No	24 (37.5%)	40 (62.5%)	
Do not know	176 (56.8%)	134 (43.2%)	

*Awareness*			
Yes	34 (28.8%)	84 (71.2%)	<0.05
No	22 (64.7%)	12 (35.3%)	
Do not know	232 (55.5%)	186 (44.5%)	

*BMI*			
Underweight	22 (84.6%)	4 (15.4%)	<0.05
Normal	152 (59.8%)	102 (40.2%)	
Overweight	78 (43.8%)	100 (56.2%)	
Obese	36 (34.5%)	68 (65.4%)	
Morbidly obese	0 (0%)	8 (100%)	

*Smoking*			
Yes	34 (73.9%)	12 (26.1%)	<0.05
No	254 (48.4%)	270 (51.5%)	

*Alcohol*			
Yes	18 (50%)	18 (50%)	0.948
No	270 (50.6%)	264 (49.5%)	

*Control BP*			
Medication	14 (20.6%)	54 (79.4%)	0.21
No medication	20 (40%)	30 (60%)	
